# Magnitude Codes for Cross-Modal Working Memory in the Primate Frontal Association Cortex

**DOI:** 10.3389/fnins.2017.00202

**Published:** 2017-04-07

**Authors:** Andreas Nieder

**Affiliations:** Animal Physiology Unit, Institute of Neurobiology, University of TübingenTübingen, Germany

**Keywords:** monkey, single neurons, prefrontal cortex, pre-SMA, frequency, number, neuronal coding

## Abstract

Quantitative features of stimuli may be ordered along a magnitude continuum, or line. Magnitude refers to parameters of different types of stimulus properties. For instance, the frequency of a sound relates to sensory and continuous stimulus properties, whereas the number of items in a set is an abstract and discrete property. In addition, within a stimulus property, magnitudes need to be processed not only in one modality, but across multiple modalities. In the sensory domain, for example, magnitude applies to both to the frequency of auditory sounds and tactile vibrations. Similarly, both the number of visual items and acoustic events constitute numerical quantity, or numerosity. To support goal-directed behavior and executive functions across time, magnitudes need to be held in working memory, the ability to briefly retain and manipulate information in mind. How different types of magnitudes across multiple modalities are represented in working memory by single neurons has only recently been explored in primates. These studies show that neurons in the frontal lobe can encode the same magnitude type across sensory modalities. However, while multimodal sensory magnitude in relative comparison tasks is represented by monotonically increasing or decreasing response functions (“summation code”), multimodal numerical quantity in absolute matching tasks is encoded by neurons tuned to preferred numerosities (“labeled-line code”). These findings indicate that most likely there is not a single type of cross-modal working-memory code for magnitudes, but rather a flexible code that depends on the stimulus dimension as well as on the task requirements.

## Multisensory innervation of the lateral PFC and premotor area pre-SMA

Brain areas supporting multimodal cognitive control functions first and foremost need input from all senses. The granular frontal lobe of the lateral prefrontal cortex (PFC) operating at the apex of the cortical perception-action hierarchy (Fuster, 2000; Miller and Cohen, 2001), receives a widespread array of converging visual and auditory afferents via two anatomically and functionally largely segregated cortical streams: a ventral and a dorsal stream (Mishkin et al., 1983; Kravitz et al., 2011), or “perception-action” pathways, respectively (Goodale and Milner, 1992).

In the visual system, the ventral occipito-temporal processing stream (via V4 and IT cortex) mediates representation of visual objects, whereas the dorsal, occipito-parietal stream (via MT/MST to the inferior parietal lobule) conveys motion information and the spatial locations of objects (Mishkin et al., 1983). In agreement with the strong visual input, the majority of PFC neurons readily show responses to sensory parameters of visual stimuli, such as color (Fuster et al., 2000), spatial location (Goldman-Rakic, 1995), motion direction (Zaksas and Pasternak, 2006), faces (O'Scalaidhe et al., 1997), or learned categories (Freedman et al., 2001).

Auditory information reaches the lateral PFC via the antero-ventral stream and the postero-dorsal stream (Rauschecker and Scott, 2009). The antero-ventral stream contains a direct projection from the anterior auditory cortex regions to the PFC (Romanski et al., 1999), and an indirect projection to the lateral and medial PFC via temporal association cortices (Medalla and Barbas, 2014). This stream shows a preference for the coding of auditory identity. The postero-dorsal stream shows a direct projection from the posterior auditory cortex regions to the PFC, and an indirect connection via the posterior parietal association cortex (Lewis and Van Essen, 2000), which in turn is connected with the PFC. This stream primarily encodes auditory space. Converging auditory input in the ventro-lateral PFC (vlPFC) gives rise to neurons that respond to the spatial and nonspatial attributes of complex auditory stimuli (Cohen et al., 2004), and represent complex acoustic stimuli such as species-specific vocalizations (Romanski and Goldman-Rakic, 2002; Averbeck and Romanski, 2006; Hage and Nieder, 2015) and sound categories (Russ et al., 2007; Lee et al., 2009).

In the somatosensory system, connections exist between the PFC with somatosensory cortical areas (Barbas and Mesulam, 1985), most notably SII (Carmichael and Price, 1995; Cipolloni and Pandya, 1999). This explains why responses to the vibration frequency of tactile stimuli are present in PFC (Romo et al., 1999; Romo and Salinas, 2003).

Directly connected to the PFC is the pre-supplementary motor area (pre-SMA) in the medial frontal lobe (Wang et al., 2005), a highly integrative brain area that plays a major role in cognition (Tanji, 2001; Hernández et al., 2002). Processed multimodal input reaches the pre-SMA via major connections from the granular PFC, as well as parts of the multimodal posterior parietal lobe (Mendoza and Merchant, 2014). As an agranular frontal lobe region, the pre-SMA is typically regarded as “premotor” area. However, unlike classical premotor areas of the frontal lobe, the pre-SMA does not have direct connections with the primary motor cortex (M1) and therefore is sometimes not considered a proper premotor area (Dum and Strick, 2002), but a higher-order area operating at a level between PFC and premotor areas (Mendoza and Merchant, 2014).

## Unimodal working memory in PFC and pre-SMA

Working memory, the ability to briefly retain and process stimuli according to task demands, is a cardinal function of the PFC. Persistent (or sustained) activity during the memory period of a delayed response task is a well known physiological correlate of working memory and particularly prominent and lasting in the PFC (Fuster and Alexander, 1971; Kubota and Niki, 1971; Goldman-Rakic, 1995) and the pre-SMA (Hernández et al., 2002; de Lafuente and Romo, 2006; Vallentin et al., 2012; Merten and Nieder, 2013; Eiselt and Nieder, 2016; Vergara et al., 2016). Delay activity in response to the memorization of individual sensory cues has been reported frequently for visual stimuli (Funahashi et al., 1989; Miller et al., 1996; Rainer et al., 1999; Rainer and Miller, 2000; Freedman et al., 2001; Merten and Nieder, 2012; Sarma et al., 2016) and during acoustic mnemonic processing (Bodner et al., 1996; Plakke et al., 2013; Plakke and Romanski, 2014). Temporally inactivating the ventrolateral PFC (vlPFC) resulted in behavioral impairment in an auditory mismatch task (Plakke et al., 2015), providing direct evidence for the vlPFC's involvement in auditory working memory. Romo et al. championed the investigation of the neural correlates of tactile working memory (Romo et al., 1999; Romo and Salinas, 2003; de Lafuente and Romo, 2006). These authors have reported that the activity of frontal lobe neurons in a working memory period is correlated with the vibration frequency of the tactile stimulus.

## Neural correlates of multisensory working memory in PFC and pre-SMA

To process stimuli that belong together, sensory information from individual objects or associated stimuli need to be integrated across modality and time. Since the largely segregated sensory pathways converge in the PFC and pre-SMA, they allow for single neurons to represent a multisensory representation. Indeed, PFC cells encoding working memory of cross-modal (audio-visual) associations have been found in monkeys trained to make associations between high/low frequency tones and red/green colors in a delayed association task (Fuster et al., 2000). Moreover, vlPFC neurons have been shown to respond during memorization of combinations of particular monkey face-voice combinations (Sugihara et al., 2006; Diehl and Romanski, 2014; Hwang and Romanski, 2015), with temporally inactivating the vlPFC through cooling resulting in a significant behavioral impairment (Plakke et al., 2015). Recently, Vergara et al. (2016) demonstrated correlates of multisensory working memory also in the pre-SMA.

## Neurons signaling magnitudes crossmodally

Depite the general finding that frontal lobe neurons have multimodal working memory properties, it remained an open question if neurons would code parametric variations of magnitude information across sensory modalities in working memory. More importantly, different types of neuronal codes may emerge based on whether relative or absolute magnitudes are to be processes (Romo and Salinas, 2003; Mendez et al., 2011; Nieder, 2016). Two recent studies combining psychophysics and electrophysiology in behaving rhesus monkey now provide insights about the cross-modal and cross-temporal code for relative and absolute magnitudes. While one study deals with relative sensory magnitude, i.e., the coding of vibrotactile frequencies (Vergara et al., 2016), the other investigates the representation of absolute numerical quantity, i.e., the number of events in a set (Nieder, 2012). Results show that neurons encode the same magnitudes based on input signals originating from different sensory cortices, but they also highlight discrepancies in the code for magnitudes.

**Figure 1:**
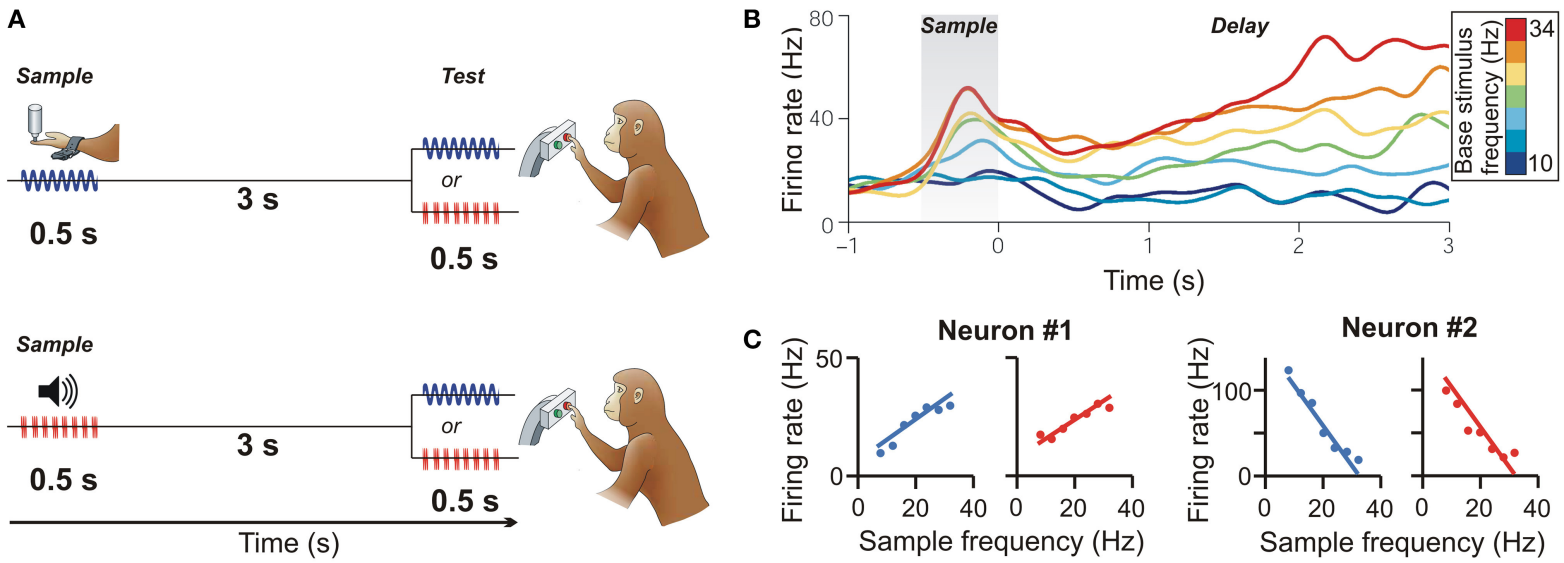
**Cross-modal representation of flutter frequency in pre-SMA. (A)** Delayed flutter discrimination task. The monkey is required to compare the frequency of two stimuli (first sample, then test) presented sequentially over a delay period between them. In the cross-modal condition, vibrotactile (*top*) or auditory flutter sample frequencies (*bottom*) are compared to auditory flutter or vibrotactile frequencies (respectively). **(B)** Time course of a PFC neuron responding monotonically to vibrotactile flutter frequencies during the sample and delay periods. Colors correspond to frequencies (Permission has been obtained from the copyright holder for the reproduction of this image from Romo and Salinas, 2003). **(C)** Monotonically increasing (neuron #1) and decreasing (neuron #2) response functions during the memory delay of two pre-SMA example neurons to both vibrotactile (*blue*) and auditory flutter frequencies (*red*). (from Vergara et al., 2016).

Vergara et al. (2016) examined how frontal lobe neurons maintain the frequency of tactile and acoustic stimuli in working memory by using a delayed discrimination task (Figure 1A). Monkeys were trained to compare the stimulus frequency across tactile and auditory modalities. In the standard task layout (Romo and Salinas, 2003), a sample stimulus vibrating at frequencies between 8 and 32 Hz was presented to the monkey's fingertip. The monkey had to remember the sample frequency during the following delay and judge whether a second test stimulus was higher or lower in frequency than the first. In another fraction of the trials, the monkey performed this task with acoustic-flutter sample stimuli. Here, sound pulses separated by silence were played, and the monkey had to judge the frequency of those acoustic flutter stimuli (Lemus et al., 2010). For instance, five sound pulses per 500 ms equaled an acoustic flutter frequency of 10 Hz. In the crucial cross-modal stimulus trials, the monkeys had to compare the frequency of a vibrotactile stimulus with the frequency of an acoustic flutter stimulus over a delay, and vice versa.

Next, the authors recorded from the pre-SMA. As described previously for PFC neurons (Romo et al., 1999), many pre-SMA neurons monotonically increased or decreased their firing rate during the delay period as a function of the vibrotactile sample stimulus frequency that had to be remembered (Figure 1B) (Hernández et al., 2002). In addition, however, the same neurons that encoded the frequency of tactile stimuli were also sensitive to the frequency of the acoustic flutter stimuli. Such bimodal neurons comprised almost 50% of task-selective pre-SMA neurons. More importantly, the response patterns of almost 50% of these neurons were congruent across modalities: bimodal neurons had similar positively or negatively monotonically increasing or decreasing responses as a function of the frequency of both the vibrotactile and acoustic stimuli (Figure 1C). As an indication that the responses of these neurons matter for the monkeys' behavior, the response strength was found to be significantly decreased whenever the monkeys made discrimination errors. This type of “summation code” seems to be the neuronal code of working memory for cross-modal flutter frequency during the higher-vs.-lower frequency discrimination task (Romo et al., 1999).

**Figure 2:**
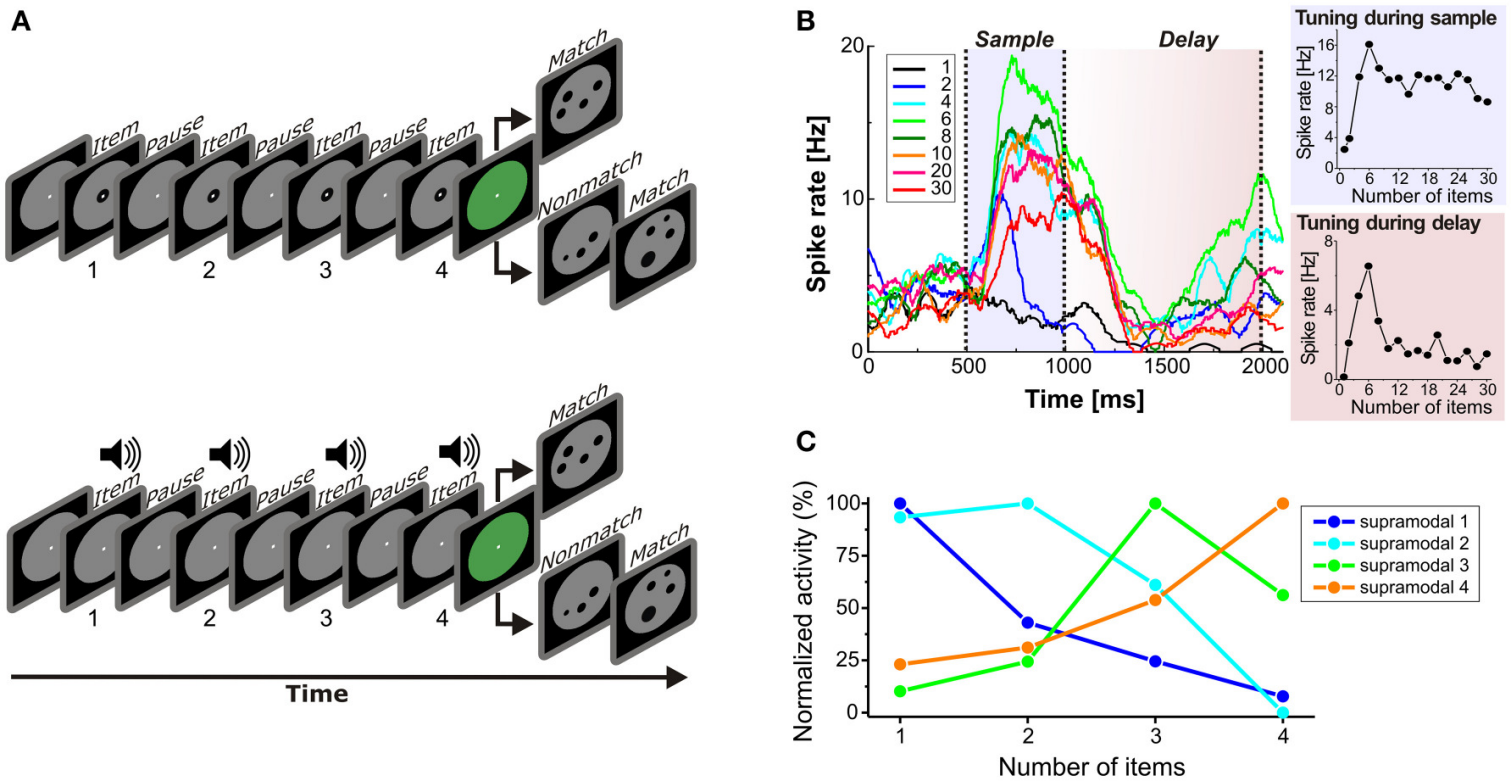
**Cross-modal representation of numerosity in PFC. (A)** Delayed match-to-numerosity task. In the sample phase, the monkey had to enumerate either visual items (*top*) or sound pulses (*bottom*), and memorize the numerosity in a delay period. After the delay, the monkey had to respond if the test dot array showed the same numerosity, and withhold response if it did not (probability 50%). In the visual trial condition, one to four dots were presented in the sample phase. In the auditory condition, one to four sound pulses were played. **(B)** Example PFC neuron (tested with visual numerosities 1 to 30) that was tuned to numerosity 6 both during sample presentation (*gray*) and memory delay (*pink*). *Left:* Spike density functions (only a selection of numbers shown for clarity). Colors correspond to specific tested numbers. *Right:* Tuning functions of this neuron during the sample (*bottom*) and delay period (*top*). (from Nieder, 2016) **(C)** Average normalized numerosity tuning functions of supramodal PFC neurons in the delay period. (from Nieder, 2012).

While the frequency of a stimulus is a sensory and continuous stimulus property, the number of items in a set (numerosity) is an abstract and discrete feature (Merten and Nieder, 2009) that needs to be encoded independent of sensory modality (“supramodal”). Three light flashes or three calls are both instances of “three.” To address the cross-modal working memory code for numerosities, monkeys were trained on a cross-modal delayed match-to-numerosity task (Figure 2A). They had to match both the number of visual dots and auditory sounds to the number of items in dot arrays within the same session (Nieder, 2012).

The monkeys were first trained with visual numerosities. One to four single dots appearing one-by-one in the sample phase were shown to the monkey while carefully controlling for temporal task factors (Nieder et al., 2006). The monkeys had to memorize the sequential number of dots during the delay period and respond if the same numerosity was displayed in the test period. Eventually, the sequential dots in the sample phase were replaced by sound pulses in half of the trials. Crucially, the monkey was now performing a cross-modal, cross-temporal numerosity matching task.

Neurons in the PFC are known to respond to the number of visual items (Nieder et al., 2002; Jacob and Nieder, 2014; Ramirez-Cardenas et al., 2016). Such numerosity-selective neurons are tuned to numerical quantity and respond with maximum discharge rates to one of the shown numerosities (the neuron's preferred numerosity) while showing progressively decreasing activity for more remote numerosities (Nieder and Merten, 2007; Figure 2B). During presentation of both visual and auditory items, individual numerosity-selective neurons in PFC were tuned to the same preferred numerosity irrespective of the modality used, i.e., supramodally. For instance, a neuron tuned to “three” responded most strongly whenever three dots or three sound in a sequence were presented. Interestingly, supramodal tuning to each of the four tested numerosities was only present in the PFC, but not yet in VIP (Nieder, 2012).

As a correlate of working memory for numerosity, many neurons were also responded to the number of items during the delay period (Nieder, 2016). Crucially, a proportion (13%) of all recorded PFC neurons was tuned to numerosity during the delay for both the visual and auditory items (Figure 2C). Whenever the monkeys made errors in judging the numerosity, the activity of the neurons to their respective preferred numerosities was significantly reduced during the delay. This suggests that the “labeled-line” delay activity of supramodal numerosity-selective cells was directly related to the monkeys' performance.

## Different magnitude codes

While crossmodal working memory for sensory and abstract magnitudes is represented by persistent delay activity of frontal lobe neurons, the codes surprisingly seem to differ. The purely monotonic response profiles characteristic for summation units that encode spectral magnitude (Vergara et al., 2016) contrast with the labeled-line code found in numerical magnitude detectors (Nieder, 2012). What factors could account for this discrepancy?

Training effects are unlikely to have caused the observed differences because the monkeys were highly trained in both the crossmodal frequency and numerosity discrimination tasks. Moreover, training does not induce a labeled-line code because tuned numerosity-selective neurons are even found in numerically-naive monkeys (Viswanathan and Nieder, 2013). Training for numerosity discrimination does increase selectivity in the PFC, but it does not change the code (Viswanathan and Nieder, 2015). Differences in the recording sites (pre-SMA vs. PFC) are also unlikely factors, given that monotonic coding of vibrotactile frequency and numerosity tuning have both been reported in the vlPFC (Romo et al., 1999; Nieder et al., 2002).

The differences in the empirical properties of the magnitudes may play a role. The number of items in a set is a discrete and highly abstract category devoid of sensory particularities (Nieder, 2016), whereas flutter frequency is a continuous and fundamentally sensory attribute of tactile and auditory stimuli (Romo et al., 1999). However, inconsistent with this interpretation is the finding that PFC neurons are also tuned to the visual spatial frequency in monkeys performing a delayed match-to-sample task (Eiselt and Nieder, 2016).

The most likely factor causing the diverging codes might therefore be the different types of neuronal magnitude representations imposed by the specific task requirements. In the flutter frequency discrimination tasks, magnitude is encoded as a relative value in relation to a reference stimulus, i.e., higher or lower than the sample frequency (Vergara et al., 2016). This relational representation might favor a summation code. This code for relative vibrotactile discrimination has been shown to rely on spike rate or a “count code” that may allow an observer to simply judge whether there are more accumulated pulses in one stimulus period than there are in the other (Luna et al., 2005).

In the numerosity studies, however, monkeys are required to encode the number of items as absolute values at a precise position on a magnitude scale, i.e., exactly 3, not more or less. Such a categorical representation favors tuned neurons, not only in the mammalian neocortex, but even in the independently and distinctly evolved avian endbrain (Ditz and Nieder, 2015, 2016). Interestingly, also tuning to absolute time intervals, another type of supramodal magnitude, was observed in pre-SMA neurons. Merchant et al. (2013) reported interval-tuned neurons that showed similar preferred intervals across modality in monkeys that performed rhythmic button pushes with variable interval durations. Such a categorical representation might favor tuned neurons, possibly by transforming a monotonous code to a labeled-line code (Verguts and Fias, 2004; Salinas, 2006). Support for this hypothesis also comes from network simulations that propose that a comparison task (larger than/smaller than) may favor summation units, whereas the match-to-sample task (same/different) may give rise to a labeled-line code (Verguts, 2007).

This assumption put forward predicts that tuned neurons would be observed in monkeys trained to judge the absolute vibrotactile frequencies in a match-to-sample task (Eiselt and Nieder, 2016). Conversely, it monkeys were to be trained on a relative numerical judgment task to indicate whether one display contains more or less items than the other, a summation code would be expected. These different codes may even alternate in individual neurons, or activate different neuronal populations, in monkeys trained to switch between an absolute magnitude match-to-sample task and a relative magnitude comparison task.

Neurons that are tuned to abstract magnitude categories might provide a computational advantage during learning of magnitude symbols in humans: they could easily be linked to arbitrary signs, such as visual shapes or auditory sounds. This is a prerequisite to establish symbolic representations of numbers through association of numerical values with numerals and number words. Indeed, neurons in the PFC of monkeys that were trained to associate the number of dots with visual shapes (for example, •^•^• with 3) responded equally well to the cardinal values in both displays (Diester and Nieder, 2007). Since the visual shapes of numerals show no systematic resemblance that can be ordered along a scale, the mapping of numerical values onto shapes would be hampered with a summation code. In a labeled-line code, however, neurons are already tuned to specific magnitudes and therefore could be easily associated with arbitrary shapes and sounds that would turn into numerical signs (Nieder, 2009).

Taken together, these contrasting findings indicate that most likely there is not a single type of working-memory code for cross-modal magnitudes. The manner in which neurons encode cross-modal magnitude information may heavily depend on the precise task at hand as well as on the stimulus dimension. This hypothesis has yet to be tested empirically with monkeys trained on both a delayed discrimination task and a delayed match-to-sample task with identical magnitude types.

## Author contributions

The author confirms being the sole contributor of this work and approved it for publication.

### Conflict of interest statement

The author declares that the research was conducted in the absence of any commercial or financial relationships that could be construed as a potential conflict of interest.
